# Impact of urea-molasses treated wheat straw levels in total mixed rations on growth and nutrient digestibility in Azikheli buffalo calves

**DOI:** 10.1007/s11250-025-04363-1

**Published:** 2025-03-10

**Authors:** Haroon Ur Rashid, Taimoor Khan, Anwar Ali Turi, Muhammad Abbas, Kalim Ullah, Isa Fusaro, Giovanni Buonaiuto, Damiano Cavallini

**Affiliations:** 1 Directorate General Livestock and Dairy Development Department, Peshawar, Pakistan; 2 Department of Veterinary Medicine, University of Teramo, Loc. Piano d’Accio, Teramo, Italy; 3 Department of Veterinary Medical Sciences (DIMEVET), Alma Mater Studiorum - University of Bologna, Ozzano Dell’Emilia, Bologna, Italy

**Keywords:** Nutrient digestibility, Feed conversion rate, Male calves, Buffalo

## Abstract

This study investigates the impact of urea molasses-treated wheat straw on growth performance and nutrient digestibility in Azikheli buffalo male calves. A longitudinal experiment was conducted on sixteen calves, randomly assigned to four experimental dietary treatments (*n* = 4 per group). The experimental diets included a control group (U0) with untreated wheat straw and three groups where urea molasses-treated wheat straw replaced 33% (U33), 66% (U66), or 100% (U100) of the straw in the total mixed ration. Results indicated that increasing the proportion of urea-treated wheat straw from 0 to 100% led to significant improvements in dry matter intake (from 2931 to 4034 g/day) and organic matter intake (from 2596 to 3623 g/day). Digestibility of dry matter, organic matter, crude protein, and crude fiber also followed an increasing trend, reaching 77.42%, 81.21%, 87.10%, and 60.22%, respectively, at the highest urea-treated wheat straw level. Furthermore, weight gain was significantly greater in calves fed 100% urea-treated wheat straw, followed by those in the U66, U33, and U0 groups. Feed conversion efficiency improved substantially in the U100 and U66 groups than U33 and U0. These findings suggest that incorporating higher levels of UMTWS in total mixed rations enhances nutrient digestibility, promoting superior growth performance and feed efficiency in Azikheli buffalo calves.

## Introduction

Globally, economic factors play a crucial role in shaping modern livestock production practices, as market fluctuations directly impact the profitability of animal farming (Assouto et al. 2020). At the same time, growing concerns over antibiotic resistance and environmental sustainability have intensified discussions on alternative feed treatments and responsible resource management (Gasparini et al. 2024). The need for sustainable farming practices has become increasingly urgent, requiring the adoption of efficient resource utilization strategies to minimize environmental impact (Lanzoni et al. 2023).

Rearing male calves is a labor-intensive and costly aspect of livestock production, with the peri-weaning period being particularly demanding. Proper management during this phase is crucial for the overall well-being of the herd. However, due to economic constraints, especially in peri-urban regions, farmers rarely invest in raising male calves (Maher et al. 2021). A common misconception among dairy farmers—exacerbated by rising milk prices—is that calf rearing is unprofitable. As a result, many farmers opt to sell milk rather than use it for feeding calves. In commercial dairy systems, most male calves are slaughtered within the first ten days of life (Buonaiuto et al. 2024). Those that are retained are typically kept with the dam to stimulate milk let-down but receive inadequate milk for proper growth and are often weaned around one year of age (Khan et al. 2007). Buffalo calves exhibit superior growth and fattening potential compared to cattle, which is attributed to their enhanced digestive efficiency (Tauqir et al. 2011). Recognizing this advantage, the Government of Pakistan has launched initiatives to promote calf rearing, particularly through calf fattening programs, incentivizing farmers to enhance meat production.

Despite these efforts, livestock nutrition in Pakistan remains suboptimal, with energy and protein levels falling significantly below animal requirements—by approximately 40% and 60%, respectively (Bibi et al. 2012). Inadequate feed resources and imbalanced conventional feeding systems are among the primary factors limiting growth rates in livestock. Due to feed shortages, animals utilize only 40–50% of their genetic potential, with forage availability meeting just 40–60% of actual requirements (Ahmad et al. 2014).

Improving forage production is fraught with challenges, including limited land availability, insufficient research resources, and unpredictable climatic conditions, particularly droughts, which reduce water availability crucial for optimal forage growth (Godde et al. 2021). Drought disrupts ecosystem stability, lowering forage yields and compromising nutritional digestibility (Ressaissi et al. 2023). Additionally, fluctuations in climate conditions, the proliferation of undesirable weeds, pest infestations, and plant diseases further diminish forage quality and productivity (Godde et al. 2021). Limited access to modern agricultural technologies and sustainable farming practices exacerbates these challenges, underscoring the need for efficient utilization of available feed resources. Traditionally, wheat straw is traditionally fed alongside green fodder to meet the dry matter requirements of ruminants (Ahmad et al. 2014).

Various feed processing techniques have been applied, either individually or in combination, to improve feed degradability, voluntary intake, and overall digestibility (Kindsigo and Kallas 2006; Mohammed et al. 2024). In developed countries, particularly in tropical regions, treatments such as urea or ammonia application (Wanapat et al. 2009) and alkali treatment (Sarwar et al. 1992; Hassan et al. 2011) have been employed to enhance the digestibility and efficiency of feed intake of dry roughages (e.g., straw; Tuen et al. 1991). Additionally, researchers have explored alternative treatments, such as urea combined with corn steep liquor (Mahr-un-Nisa et al. 2004) and organic acid applications (Sarwar et al. 2004). Through solid-state fermentation, these feedstuffs provide more fermentable substrates within the rumen, improving digestion and nutrient assimilation (Shahzad et al. 2016).

Compared to untreated wheat straw, treated roughages exhibit improved palatability, higher nutritive value, and increased daily feed intake, contributing to better overall animal health (Kashongwe et al. 2014). Sarwar et al. (2011) and Nisa et al. (2007) demonstrated that urea-treated wheat straw could replace up to 30% of dietary concentrate without compromising productive performance. Similarly, Mohammed et al. (2024) reported that the inclusion of urea-lime-treated wheat straw, when supplemented with concentrate at 1.2% of live weight, resulted in significant growth improvements in crossbred calves while remaining a cost-effective feeding strategy.

Treating wheat straw with urea and ammonium bicarbonate has been reported to increase crude protein levels from 3.2% (untreated) to 8.7% and 9.5%, respectively (Ali et al. 2002). Additionally, feeding urea-treated straw has been shown to reduce the need for concentrate supplementation by increasing nitrogen availability and enhancing palatability, digestibility, and weight gain, particularly during feed shortages (Abate and Melaku 2009).

This study aims to evaluate the effects of incorporating urea-molasses-treated wheat straw into the diet of Azikheli buffalo male calves. Specifically, it examines the impact of this dietary intervention on growth performance, dry matter intake (DMI), nutrient digestibility, and body weight gain.

## Material and methods

The trial occurred at the Livestock Research and Development Station in Dir Lower, KPK, Pakistan. Ethical approval for this study was granted by the Animal Welfare and Care Committee of the Livestock and Dairy Development Department, on November 3, 2020, under protocol number 07022024. The experiment was conducted by established guidelines to ensure minimal discomfort to the animals, and proper management practices were employed throughout the research process. All research activities involving animals were carried out in compliance with relevant state and local laws and regulations.

Sixteen pre-weighed, six-month-old male Azikheli buffalo calves, an indigenous breed (*Bubalus bubalis*; Khan et al. 2013), were selected for the study. At the start of the experiment, the average body weight of the calves was 70.2 ± 0.5 kg (mean ± SD). The study was conducted over a period of three months. The first 15 days of the trial served as an adaptation period, allowing the animals to adjust to the experimental conditions. Thereafter, every alternate 10 days were designated as collection periods for data gathering. The calves were randomly assigned to one of four dietary treatment groups. The control group received a diet with no urea-molasses-treated wheat straw (0U). In the second treatment, 33% of the wheat straw was replaced with urea molasses-treated straw (33U), while in the third treatment, this proportion increased to 66% (66U). In the fourth treatment, wheat straw was entirely replaced with urea-molasses-treated straw (100U). The composition of the experimental diets is detailed in Table 1.

**Table 1:** Ingredients and chemical composition of experimental rations

Ingredients, % on DM basis	U0^1^	U33^2^	U66^3^	U100^4^
Cotton seed cake	7.0	7.5	7.5	7.6
Canola meal	10.0	7.5	7.0	5.0
Wheat straw	30.0	20.1	10.2	0.0
Urea-treated wheat straw	0.0	9.9	19.8	30.0
Corn Silage	20.0	20.5	21.0	21.5
Corn grain^2^	8.0	9.0	10.3	11.5
Rape seed cake	7.0	5.5	5.0	3.0
Wheat bran	11.5	13	12.7	14.5
Molasses	5	5.5	5.5	6.0
Mineral mix	0.5	0.5	0.5	0.5
Salt	0.5	0.5	0.5	0.5
Urea	0.5	0.0	0.0	0.0
**Parameters**^**3**^				
DM, %	85.8	84.7	82.3	81.6
CP, %DM	12.1	12.9	13.1	13.2
CF, %DM	26.7	26.8	25.3	22.7
Ash, %DM	11.4	10.8	10.4	10.2

^1^U0: Urea-treated straw is 0% of the total straw; U33: Urea-treated straw is 33% of the total straw; U66: Urea-treated straw is 66% of the total straw; U100: Urea-treated straw is 100% of the total straw

^2^Below the EU maximum tolerable level (Girolami et al. 2022)

^3^*DM* Dry matter, *CP* Crude Protein, *CF* Crude Fiber

To prepare the urea molasses-treated straw, a solution containing 4% molasses and 4% urea on a fed basis was applied to the wheat straw. The treated straw was then packed in cement pits and left for approximately 40 days to allow for proper fermentation, following the method described by Ali et al. (1993). Experimental diets were formulated as total mixed rations (TMRs) daily, adhering to the crude protein (CP) and energy requirements recommended by NRC (2001). Each diet was balanced to provide similar concentrations of energy and CP on a dry matter (DM) basis.

Before the start of the experiment, all equipment and metabolic cages were thoroughly cleaned and disinfected. Upon the start, each buffalo calf was individually weighed and placed in a randomly selected metabolic cage. A preliminary adaptation period of 15 days was provided to all animals before the start of the 75-day experiment. Diets were offered ad libitum twice daily (at 8 am and 4 pm), with unrestricted access to clean, fresh water.

Feed and fecal samples were collected daily throughout the experimental period. Representative samples of both offered and refused feed were stored in clean, labeled polythene bags and immediately frozen to prevent moisture loss. The total fecal collection was conducted over 24 h, and representative samples were similarly stored for subsequent analysis.

The chemical composition of the feed and fecal samples, including dry matter (DM; method 930.15), crude protein (CP; method 976.05), crude fiber (CF; method 962.09), and ash (method 942.05), was analyzed according to the methods outlined by the Association of Official Analytical Chemists (AOAC 1990), as described in previous studies (Buonaiuto et al. 2021; Felini et al. 2024).

Feed intake was recorded daily, while body weight was measured every 14 days according to the methods described by Cavallini et al., (2023). Daily dry matter intake (DMI) was determined as the difference between feed offered and feed refused. Body weight gain (BWG) was calculated as the difference between the final and initial body weights. The feed conversion ratio (FCR) was obtained by dividing total feed intake by body weight gain. Additionally, nutrient intake was assessed for organic matter (OMI), crude protein (CPI), and crude fiber (CFI). Digestibility coefficients were determined based on total nutrient intake and fecal excretion.

### Statistical analysis

The data obtained were analyzed using a Complete Randomized Design for each parameter (feed intake, digestibility, weight gain, and Feed Conversion Ratio (FCR)) with statistical software (JMPpro v17.1). A mixed model for repeated measures was applied, with dietary treatments as the fixed effect and each subject as the experimental unit. Means were differentiated using Tukey’s adjusted P-values when a significant F-test (*P* ≤ 0.05) was detected. Residuals were then assessed for normality.

## Results

Table 1 reports the chemical composition of the feedstuff, illustrating variations in nutrient content across the diets. As the inclusion of urea-treated wheat straw increased, there was a decrease in the dry matter (DM), crude fiber (CF), and ash content of the diets, while the crude protein (CP) content increased.

Nutrient intake, fecal output, and digestibility metrics are reported in Table 2. Significant differences (*p* < 0.01) were observed across the different rations. The DMI ranged from 2931 g in U0 to 4034 g in U100, with a consistent increase observed with higher urea-treated straw levels. The OMI and CPI followed similar trends, with OMI increasing from 2596.3 g in U0 to 3622.9 g in U100, and CPI from 355.5 g in U0 to 530.5 g in U100. The CFI also increased from 781.0 g in U0 to 914.7 g in U100. Fecal output showed corresponding increases, with DM output rising from 2074.9 g in U0 to 3123.0 g in U100, and OM output from 1934.2 g in U0 to 2942.0 g in U100. The CP and CF fecal outputs increased with higher levels of urea-treated straw, indicating enhanced digestion and nutrient absorption. Digestibility measures improved significantly with increased urea treatment. The DMD rose from 70.8% in U0 to 77.4% in U100. The OMD increased from 74.5% to 81.2%, CPD from 79.4% to 87.1%, and CFD from 51.1% to 60.2% (Table 2).

**Table 2:** Nutrient intake, fecal output and digestibility of Azikhli buffalo male calves fed TMR^1^ containing different levels of fermented wheat straw

Parameters	U0^1^	U33		U66	U100	SEM	*p*-value
Intakes^2^							
DMI, g	2931.0^d^	2989.0^c^		3349.0^b^	4034.0^a^	13.1	< .01
OMI, g	2596.3^d^	2665.6^c^		2999.7^b^	3622.9^a^	11.7	< .01
CPI, g	355.5^d^	385.6^c^		439.4^b^	530.5^a^	1.7	< .01
CFI, g	781.0^d^	799.8^c^		847.7^b^	914.7^a^	2.1	< .01
Fecal output							
DM, g	2074.9^d^	2189.5^c^		2503.7^b^	3123.0^a^	12.3	< .01
OM, g	1934.2^d^	2048.9^c^		2371.5^b^	2942.0^a^	10.1	< .01
CP, g	282.4^d^	316.9^c^		371.0^b^	462.0^a^	1.3	< .01
CF, g	399.4^d^	443.4^c^		491.8^b^	550.8^a^	3.2	< .01
Digestibility^3^							
DMD, %DM	70.8^d^	73.3^c^		74.8^b^	77.4^a^	0.3	< .01
OMD, %OM	74.5^d^	76.9^c^		79.1^b^	81.2^a^	0.2	< .01
CPD, % CP	79.4^d^	82.2^c^		84.4^b^	87.1^a^	0.2	< .01
CFD, %CF	51.1^d^	55.5^c^		58.0^b^	60.2^a^	0.4	< .01

Means in the same row with different superscripts are significantly different (*P* < 0.05)

^1^U0: Urea-treated straw is 0% of the total straw; U33: Urea-treated straw is 33% of the total straw; U66: Urea-treated straw is 66% of the total straw; U100: Urea-treated straw is 100% of the total straw

^2^*DMI* dry matter intake, *OMI* organic matter intake, *CPI* crude protein intake, *CFI* crude fiber intake

^3^*DMD* dry matter digestibility, *OMD* organic matter digestibility, *CPD* crude protein digestibility, *CFD* crude fiber digestibility

The body weight evolution of the calves is detailed in Table 3. Initial body weights were similar across all groups, averaging around 70 kg. Final body weights, however, showed significant differences (*p* < 0.01), with the U100 group achieving the highest final body weight of 117.5 kg, compared to 106.0 kg in the U0 group. The BWG also reflected this trend, increasing from 35.8 kg in U0 to 47.1 kg in U100. Finally, the FCR, showed a slight but significant increase with higher urea-treated straw levels, ranging from 7.2 in U0 to 7.7 in U100 (Table 3).

**Table 3:** Body weight evolution of Azikhli buffalo male calves fed TMR^1^ containing different levels of fermented wheat straw

Parameters^2^	U0^2^	U33^3^	U66^4^	U100^5^	SEM	*p*-value
Initial BW, Kg	70.3	69.4	70.6	70.4	0.7	0.66
Final BW, Kg	106.0^c^	107.6^bc^	111.0^b^	117.5^a^	1.1	< .01
BWG, Kg	35.8^d^	38.2^c^	40.4^b^	47.1^a^	0.4	< .01
FCR (Kg DMI/Kg BWG)	7.2^c^	7.2^bc^	7.5^ab^	7.7^a^	0.01	< .01

Means in the same row with different superscripts are significantly different (*P* < 0.05)

^1^U0: Urea-treated straw is 0% of the total straw; U33: Urea-treated straw is 33% of the total straw; U66: Urea-treated straw is 66% of the total straw; U100: Urea-treated straw is 100% of the total straw

^2^*BW* Body weight, *BWG* Body weight gain, *FCR* Feed conversion rate

## Discussion

This study evaluates the impact of urea molasses-treated wheat straw on the growth performances, body weight gain, dry matter intake and nutrient digestibility in Azikheli buffalo male calves. The research is conducted within the broader context of addressing challenges and improving the efficiency of livestock production in Pakistan.

Increase in crude protein (CP) content of treated wheat straw due to urea treatment aligns with findings by Mesfin and Ledin (2004). Similarly, Agrawal et al. (1989) observed that treated rice straw increased CP from 3.94% to 12.96%, while treated wheat straw increased CP from 3.43% to 7.83%. They also noted an improvement in CP content with urea-treated paddy straw. This increase in CP content may be linked to wheat straw's response to urea treatment, supported by Chenost and Kayouli (1997), who found that straw responds favorably to urea treatment due to its lower nutrient content. Another explanation is that the increase in nitrogen content transferred from urea to the straw enhances the resulting CP. In this research, diets were balanced by adding urea to the TMR ration (U0) to address this issue.

Regarding the intake results, our findings support those of Yulistiani et al. (2003), who observed significantly increased voluntary DMI per kg of metabolic weight in sheep when 4% urea-treated straw was used. MacDearmid et al. (1988), Sethy et al. (2016), and Sarwar et al. (2006) found increased DMI, OMI, CPI, and CFI in buffalo calves fed treated wheat straw containing 4% urea and 4% molasses. Gunun et al. (2013) also noted improvements in feed intake, rumen fermentation, and efficiency of microbial nitrogen synthesis when treated rice straw was fed to cross-bred dairy steers.

Regarding body condition, our findings align with Hoque et al. (2019), who reported the highest BWG in the TMR-fed group with urea-molasses-treated straw. Emmanuel et al. (2015), and Hassoun et al. (2002) obtained results correlated with our study in BWG. Hoque et al. (2019) also reported significantly higher FCR for treated paddy straw compared to the control group. Our findings also support Rahman et al. (2009), who reported better FCR in emaciated bulls due to the supplementation of molasses and urea-treated straw.

Addressing challenges related to limited feed resources and imbalanced feeding systems is crucial for maximizing livestock productivity. Future research should prioritize optimizing feed resources, enhancing feed quality, and improving nutrient utilization to promote better growth performance in Azikheli buffalo. Given constraints like limited land availability and climate change impacting forage production, innovative approaches to enhance forage production are imperative. Exploring novel methods of treating roughages, such as urea or alkali treatment as applied in this study, holds promise for enhancing digestibility and nutrient assimilation in animals. Future research could refine these treatment methods or explore novel approaches to further bolster feed digestibility and animal growth potential. Finally, disseminating the results of this research, as outlined in recent, and integrating novel findings into the curricula of veterinary and animal science courses (Muca et al. 2023) are crucial steps in advancing livestock management practices.

In conclusion, the complete replacement of wheat straw with urea-molasses-treated straw (100U) proved to be the most effective treatment, leading to the highest nutrient digestibility, improved feed conversion efficiency, and a significant increase in calf growth rate. These findings suggest that maximizing the inclusion of urea-molasses-treated straw in TMR formulations can optimize performance in young animals. Further studies are recommended to assess the long-term effects of this feeding strategy on the productive and reproductive performance of adult animals.

## Data Availability

None of the data were deposited in an official repository and are available upon request.
